# The effects of kinesio taping on dynamic balance in patients with chronic ankle instability: a systematic review and meta-analysis

**DOI:** 10.3389/fphys.2026.1823162

**Published:** 2026-04-27

**Authors:** Shilong Meng, Xinlei Fu, Yawei Xu, Xu Zhang, Zhiliang Peng, Yanguang Cao, Binbin Tang, Xiaolin Shi, Kang Liu, Jiepeng Zhu

**Affiliations:** 1 The Second Clinical Medical College of Zhejiang Chinese Medical University, Hangzhou, Zhejiang, China; 2 The First Affiliated Hospital of Zhejiang Chinese Medical University, Hangzhou, Zhejiang, China; 3 The Second Affiliated Hospital of Zhejiang Chinese Medical University, Hangzhou, Zhejiang, China

**Keywords:** chronic ankle instability, dynamic balance, kinesio taping, meta-analysis, sports injuries

## Abstract

**Background:**

Kinesio taping (KT) is commonly used as an adjunct in the rehabilitation of chronic ankle instability (CAI) and is proposed to enhance proprioception and dynamic stability. However, evidence regarding its clinical effectiveness remains inconsistent. This systematic review and meta-analysis aimed to quantify the effects of KT on dynamic balance and related functional outcomes in individuals with CAI.

**Methods:**

We systematically searched PubMed, Web of Science, Embase, the Cochrane Library, CNKI, Wanfang Data, and the VIP Database, covering the period from the inception of each database to 1 October 2025. Randomized controlled trials or randomized crossover trials involving KT intervention for CAI patients were included. Outcome measures included Y-balance test (YBT) scores (anterior, posteromedial, posterolateral), Star Excursion Balance Test (SEBT) scores (anterior, posteromedial, posterolateral), and Single Hop Distance Test (SHDT). The Cochrane Risk of Bias tool was used to assess study quality, and the certainty of evidence for the outcomes was evaluated using the GRADE approach. Meta-analysis was conducted using Stata 18.0 software, with results presented as standardized mean differences (*SMD*) and their 95% confidence intervals (*95% CI*).

**Results:**

A total of 8 studies involving 190 participants from 4 countries/regions were included. Compared with the control group, the KT group showed a small but significant improvement in the YBT total score (*SMD* = 0.269, *95% CI*: 0.031-0.508), YBT posterolateral score (*SMD* = 0.385, *95% CI*: 0.106-0.664), and SEBT anterior score (*SMD* = 0.686, *95% CI*: 0.083-1.289), suggesting that KT may exert a modest beneficial effect on dynamic balance in certain directions. However, no statistically significant differences were observed between groups for SEBT total score, posteromedial and posterolateral directions, or SHDT (*P*>0.05).

**Conclusion:**

Current evidence suggests that KT may provide a modest, direction-specific improvement in dynamic balance in individuals with CAI, while additional benefits for functional performance remain uncertain. KT may be considered a simple and low-cost adjunct to comprehensive rehabilitation (e.g., strengthening, proprioceptive, and balance training), but should not replace conventional interventions. Further high-quality trials with standardized taping protocols and medium- to long-term follow-up are needed.

**Systematic review registration:**

https://www.crd.york.ac.uk/prospero/, identifier CRD420251165413.

## 1 Introduction

Chronic ankle instability (CAI) is a common long-term sequela of ankle trauma. It is characterized by recurrent ankle sprains and perceived instability, often accompanied by pain and swelling. CAI can compromise athletic performance and daily mobility, thereby exerting sustained adverse effects on quality of life as well as physical and psychological well-being (Gribble et al., 2013; Chang et al., 2021). According to the International Ankle Consortium consensus statement, although the direct cost of treating a single ankle sprain is relatively low, long-term functional impairment and recurrent injuries can substantially increase the overall medical and socioeconomic burden; the annual healthcare expenditure related to ankle injuries has been estimated to reach billions of dollars (Gribble et al., 2016). Functionally, the ankle joint plays a pivotal role in weight-bearing and postural control. Following a sprain, patients are at increased risk of recurrent injury and may develop gait alterations and reduced sports performance (Delahunt et al., 2018). Notably, impaired dynamic balance is considered a key functional feature of CAI and is closely linked to deficient postural control and re-injury risk, making it a critical target in rehabilitation.

Current management of CAI primarily relies on conservative rehabilitation approaches, including exercise therapy, proprioceptive training, balance and coordination training, neuromuscular control training, and biofeedback. Evidence suggests that these interventions can improve motor function and activities of daily living and may enhance balance and postural control to some extent (Kaminski et al., 2013; Rendos et al., 2017; Rivera et al., 2017; Dhillon et al., 2023). However, many programs require specialized facilities or equipment, supervision by experienced clinicians, prolonged treatment duration, and high patient adherence. These barriers may limit accessibility and reduce the likelihood that some patients complete a full course of rehabilitation, which can, in turn, attenuate clinical benefits. Therefore, rehabilitation strategies that are safe, cost-effective, easy to implement, and scalable are of clear clinical and public health value. Such approaches may help reduce secondary injury risk, facilitate functional recovery, and lessen the burden on families and society.

Kinesiology tape is an elastic, adhesive, and breathable tape, and its application is commonly referred to as Kinesio taping (KT). When applied to the skin, KT is proposed to provide continuous cutaneous and proprioceptive stimulation, potentially modulating muscle activation patterns and neuromuscular timing. It may also support local circulation and provide external support without substantially restricting joint mobility (Expert Consensus Group on the Clinical Application of Kinesio Taping in China, 2021). In recent years, KT has been widely adopted in sports medicine and rehabilitation because it is simple, non-invasive, and relatively low-cost (Williams et al., 2012; Tran et al., 2023). Several studies suggest that KT may improve neuromuscular control, proprioception, and balance in individuals with CAI (Simon et al., 2014; Jackson et al., 2016). Nevertheless, findings remain inconsistent. For example, Nunes et al. (2021) reported limited effects of KT on functional or athletic performance in individuals with or without ankle injuries, and Raymond et al. (2012) observed no significant improvement in proprioceptive acuity among patients with functional ankle instability. Collectively, the clinical value of KT for CAI remains controversial.

At present, international research has increasingly examined KT in ankle injuries and functional recovery. However, previous systematic reviews often included heterogeneous populations (e.g., healthy athletes, acute ankle sprains, and other lower-limb conditions) and primarily focused on composite outcomes such as subjective functional scores or athletic performance (Biz et al., 2022; Yang et al., 2022). Quantitative synthesis targeting dynamic balance—one of the most relevant functional outcomes in CAI—remains limited. Meanwhile, multiple randomized controlled trials, including randomized crossover trials, have been published in recent years, providing an updated evidence base suitable for meta-analysis. Therefore, this study was designed to address the following research question: In individuals with chronic ankle instability, does Kinesio taping, compared with sham taping or no taping, improve dynamic balance and related functional outcomes? To answer this question, we systematically reviewed and synthesized the available evidence.

## 2 Methods

This study was conducted and reported in accordance with the Preferred Reporting Items for Systematic Reviews and Meta-Analyses (PRISMA) 2020 statement (Page et al., 2021). The research protocol has been registered on the PROSPERO platform. Registration number is CRD420251165413.

### 2.1 Retrieval strategy

We systematically searched PubMed, Web of Science, Embase, Cochrane Library, CNKI, Wanfang Data, and VIP Database, covering the period from each database’s inception to 1 October 2025. The search strategy employed a combination of “subject headings + free-text terms” with no language restrictions. Chinese search terms included: “Chronic Ankle Instability, Ankle Instability, CAI” and “Kinesio Taping, Kinesiotape, Kinesiology tape, KT, Tape”; English search terms comprised: “Chronic Ankle Instability, Ankle Instability, CAI” and “Kinesio Taping, Kinesiotape, Kinesiology tape, KT, Tape”. Additionally, we manually reviewed reference lists of relevant literature to supplement studies not identified by database searches, and conducted further screening of selected publications.

**Figure 1:**
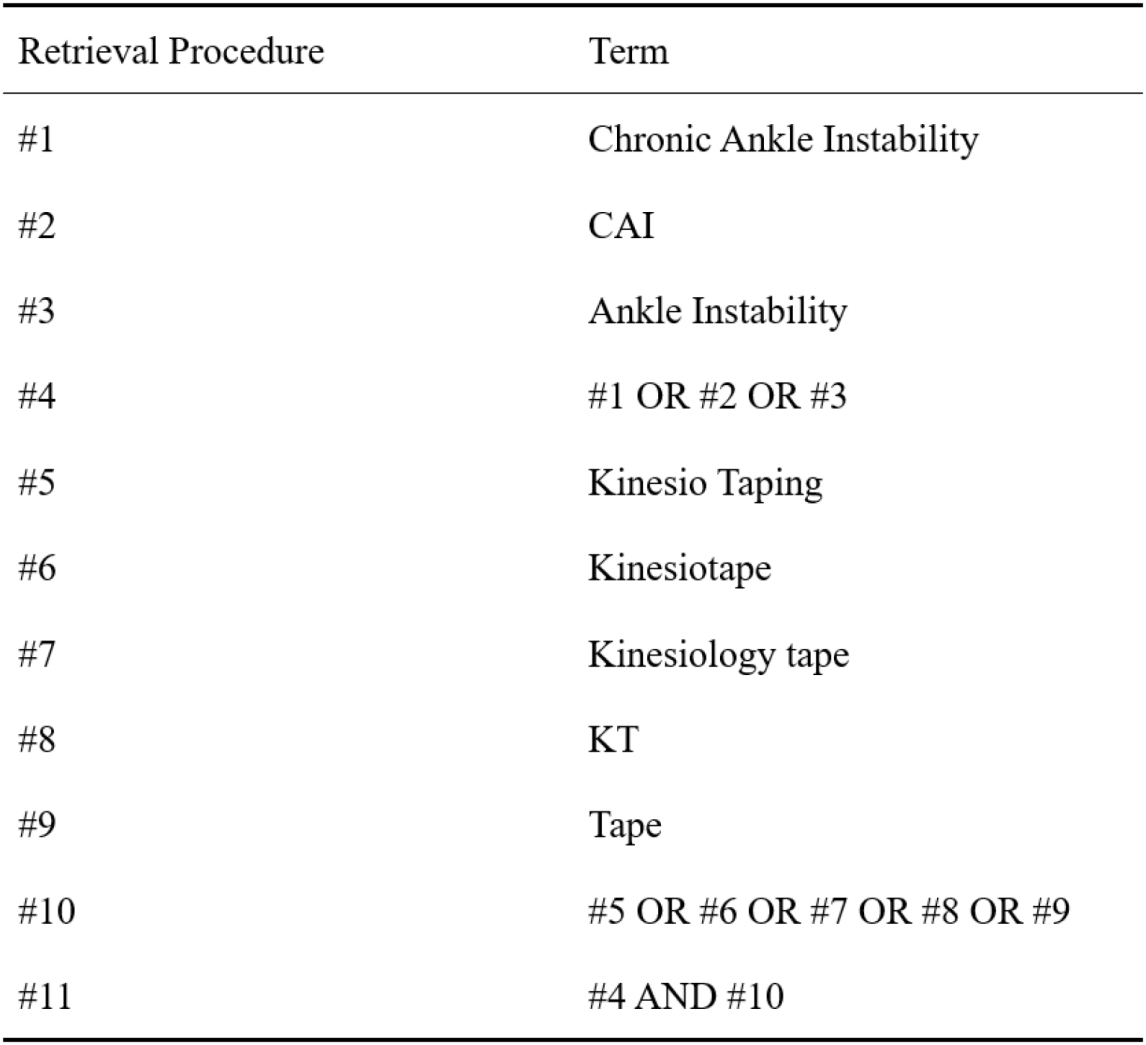
PubMed retrieval procedure flowchart.

Taking PubMed as an example, the literature retrieval strategy can be formulated as follows: ((Chronic Ankle Instability [Title/Abstract] OR Ankle Instability [Title/Abstract] OR CAI [Title/Abstract]) AND (Kinesio Taping [Title/Abstract] OR Kinesiotape [Title/Abstract] OR Kinesiology tape [Title/Abstract] OR KT [Title/Abstract] OR Tape [Title/Abstract])). For specific steps on searching PubMed, see **Figure 1**. The full search strategies for all databases are provided in **Supplementary File 1**.

### 2.2 Inclusion and exclusion criteria

The initial screening and inclusion of literature were based on the PICOS framework. Studies meeting the following criteria were considered eligible for inclusion:

*P (Population):* Participants were patients diagnosed with CAI as defined by the original studies, with no restrictions on sex, age, or occupation. We preferentially included studies adopting the diagnostic criteria recommended by the International Ankle Consortium, which include: (1) a history of at least one significant lateral ankle sprain; (2) recurrent episodes of “giving way” and/or perceived instability; and (3) CAI confirmed using validated functional/symptom questionnaires and/or clinical functional assessments (Gribble et al., 2013). Studies using other diagnostic approaches were also eligible, provided that the criteria were clearly described and clinically consistent with CAI. For studies including both CAI and healthy participants, only data from the CAI cohort were extracted; healthy control data were not synthesized.

*I (Intervention):* The experimental group received KT as the only taping intervention. Taping could be applied to structures around the ankle joint and/or lower-limb muscle groups relevant to ankle stability. Co-interventions were permitted only if applied equally to both groups.

*C (Comparison):* The control group did not receive KT and could receive no taping or placebo/sham taping (e.g., non-tension or non-functional taping). Studies were excluded if the control group received additional functional ankle taping methods.

*O (Outcome):* The primary outcomes were dynamic balance measures, including the Y-Balance Test (YBT) scores. Secondary outcomes included Star Excursion Balance Test (SEBT) scores and the Single Hop Distance Test (SHDT). Studies were required to report extractable quantitative data for at least one eligible outcome.

*S (Study design):* Only randomized controlled trials (parallel-group) and randomized crossover trials were eligible. For crossover trials, randomization of condition order and an adequate washout period (or justification of no carryover) were required. Non-randomized studies, laboratory repeated-measures studies without randomization, exploratory studies, and pre–post designs without a concurrent control/sham condition were excluded.

Exclusion criteria: (1) duplicate publications or studies; (2) case reports, conference abstracts, reviews, and non-randomized studies; (3) studies without a control condition, or with missing key outcome data that could not be obtained from the authors; and (4) studies with insufficient methodological information to permit risk-of-bias assessment and with no response after attempts to contact the authors.

### 2.3 Literature screening and data extraction

Study selection: All retrieved records were imported into EndNote X9.1, and duplicates were removed. Two reviewers (Zhang and Xu) independently screened titles and abstracts to identify potentially eligible studies. Full texts were then obtained and assessed independently against the PICOS framework and the predefined exclusion criteria. Any disagreements were resolved through discussion; if consensus could not be reached, a third reviewer (Liu) adjudicated.

Data extraction: Two reviewers (Zhang and Xu) independently extracted data using a predesigned standardized form. Extracted information included: (1) study characteristics (first author, publication year, country/region, study design, sample size, and participant demographics); (2) intervention details (protocols for the KT and control conditions, including taping location(s), application method, duration, comparator type, and follow-up time points, if applicable); (3) outcome data (means, standard deviations, and sample sizes at the prespecified post-intervention time point(s), or other statistics convertible to effect sizes); and (4) methodological information required for risk-of-bias assessment. After extraction, the two reviewers cross-checked all entries, and discrepancies were resolved by a third reviewer (Liu).

For studies with incomplete information or outcomes reported only in figures, we contacted the corresponding authors to request additional data. If the required data could not be obtained, the study was not included in the quantitative synthesis for the relevant outcome.

### 2.4 Literature quality assessment

The methodological quality of the included studies was assessed by evaluating risk of bias using the original Cochrane Risk of Bias tool (RoB 1.0) (Higgins et al., 2011). The following domains were assessed: random sequence generation (selection bias), allocation concealment (selection bias), blinding of participants and personnel (performance bias), blinding of outcome assessment (detection bias), incomplete outcome data (attrition bias), selective reporting (reporting bias), and other potential sources of bias (e.g., baseline imbalance or, for crossover trials, potential period/carryover effects). Each domain was judged as low risk, unclear risk, or high risk. Two reviewers independently performed the assessments, and discrepancies were resolved through discussion; if consensus could not be reached, a third reviewer adjudicated.

### 2.5 Certainty of evidence assessment

The certainty of evidence for the outcomes was assessed using the GRADE approach (Guyatt et al., 2008). Because the included studies were randomized controlled trials or randomized crossover trials, the certainty of evidence was initially rated as high and was downgraded, when appropriate, based on risk of bias, inconsistency, indirectness, imprecision, and publication bias. The overall certainty for each outcome was rated as high, moderate, low, or very low. Two reviewers independently assessed the certainty of evidence, and disagreements were resolved through discussion or consultation with a third reviewer when necessary.

### 2.6 Statistics

Statistical analyses were performed using Stata (version 18.0). Given that included studies reported outcomes using different scales and/or units, continuous outcomes (e.g., YBT and SEBT) were synthesized as standardized mean differences (*SMDs*) with 95% confidence intervals (*CIs*). When necessary, other summary statistics were transformed into *SMDs* using standard methods.

When multiple post-intervention time points were reported, data were extracted at a prespecified time point to improve comparability across studies. Specifically, we prioritized the earliest post-intervention assessment (immediately post-intervention) to capture short-term effects and to minimize heterogeneity arising from different follow-up durations. If an immediate assessment was unavailable, the closest subsequent time point was used.

Between-study heterogeneity was assessed using the Cochran *Q* test and the *I²* statistic. A random-effects model was used when heterogeneity was considered substantial (e.g., *I²* > 50% and/or *Q*-test *P* < 0.10); otherwise, a fixed-effect model was applied (Higgins and Thompson, 2002). Subgroup analyses were conducted by reach direction (anterior, posteromedial, posterolateral) for YBT and SEBT to explore potential clinical heterogeneity.

Sensitivity analyses for the primary outcome (YBT) were conducted using a leave-one-out approach. Publication bias was explored by visual inspection of funnel plots for the primary outcome only; formal tests (*Egger’s* or *Begg’s*) were not performed due to the small number of studies. All tests were two-sided, and *P* < 0.05 was considered statistically significant.

For randomized crossover trials, outcomes were extracted as condition-specific means and SDs as reported; paired-difference statistics or within-subject correlation coefficients were not consistently available, and results were interpreted with caution.

## 3 Results

### 3.1 Literature screening process and outcomes

**Figure 2:**
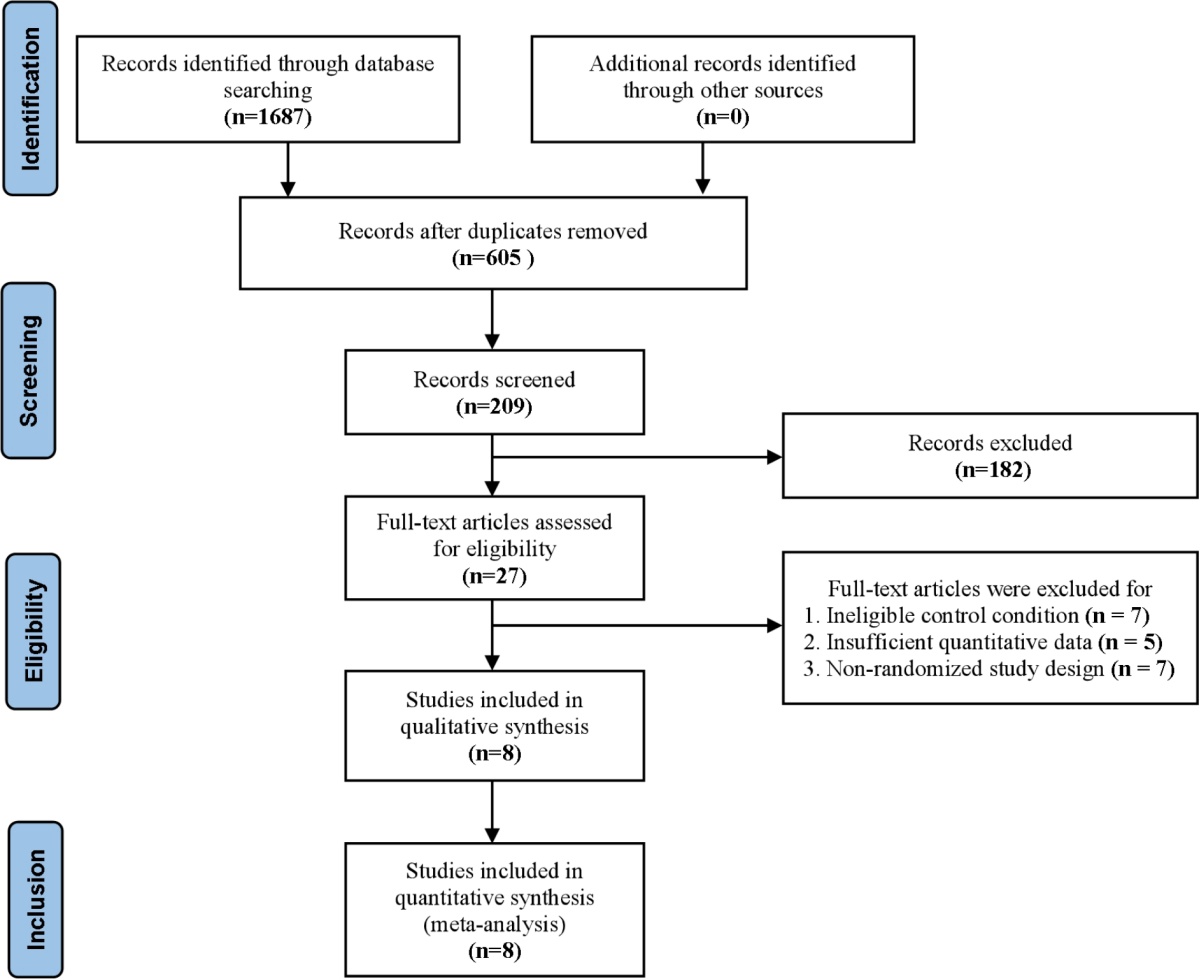
PRISMA flow diagram.

The database search identified 1, 687 records, and 605 unique records remained after duplicate removal. After title and abstract screening, 27 full-text articles were assessed for eligibility. Of these, 19 were excluded for predefined reasons, including ineligible control conditions, insufficient quantitative outcome data for meta-analysis, and non-randomized study designs. Ultimately, 8 studies (Lee and Lee, 2015; Hadadi et al., 2020; Yin, 2020; Yu, 2021; Safari et al., 2023; Wang et al., 2023; Harry-Leite et al., 2024; Yuan et al., 2025) involving 190 participants were included in the final analysis. The study selection process is summarized in the PRISMA flow diagram (**Figure 2**).

### 3.2 Basic characteristics of included literature

A total of eight studies from four countries/regions were included, comprising 190 participants with CAI. Regarding outcome reporting, six studies (Yin, 2020; Yu, 2021; Safari et al., 2023; Wang et al., 2023; Harry-Leite et al., 2024; Yuan et al., 2025) reported Y-Balance Test (YBT) outcomes, two studies (Lee and Lee, 2015; Hadadi et al., 2020) reported Star Excursion Balance Test (SEBT) outcomes, and two studies (Hadadi et al., 2020; Safari et al., 2023) reported the Single Hop Distance Test (SHDT); some studies reported more than one outcome. The general characteristics of the included studies are summarized in **Table 1**, and additional study details are provided in **Table 2**.

**Table 1:** Characteristics of included studies and participants.

Number	Study	Country/regions	Study design	Population	Intervention		Sample size		Outcome
					T	C	T	C	
1	Lee and Lee (2015)	South Korea	Randomized crossover trial	Football players with CAI	KT	PT	9	9	SEBT
2	Hadadi et al. (2020)	Iran	Randomized controlled trial	Patients with CAI	KT	PT	19	19	SEBT SHDT
3	Yin (2020)	China	Randomized crossover trial	Patients with CAI	KT	PT	35	35	YBT
4	Yu (2021)	China	Randomized crossover trial	Patients with CAI	KT	NT	15	15	YBT
5	Safari. et al, 2021 (Safari et al., 2023)	Iran	Randomized controlled trial	Volleyball players with CAI	KT	PT	13	13	YBT SHDT
6	Wang et al. (2023)	China	Randomized crossover trial	Patients with CAI	KT	NT	16	16	YBT
7	Harry-Leite et al. (2024)	Portugal	Randomized controlled trial	Football players with CAI	KT	PT	23	23	YBT
8	Yuan et al. (2025)	China	Randomized crossover trial	Patients with CAI	KT	PT	15	15	YBT

T, Test group; C, Control group; KT, Kinesio taping; PT, Placebo taping; NT, No taping; CAI, Chronic ankle instability; SEBT, Star Excursion Balance Test; YBT, Y-Balance Test; SHDT, Single Hop Distance Test. For randomized crossover trials, the sample size refers to the total number of participants (within-subject design).

**Table 2:** Intervention and outcome characteristics of included studies.

Study	Diagnostic criteria	Detailed intervention measures		Assessment time	Study conclusion
		T	C		
Lee and Lee (2015)	History of untreated severe ankle sprain; Ankle instability or fluctuating; CAIT score ≤ 27.	The first strip was applied in two directions from the talus to the calcaneus. The second strip commenced 5 cm above the medial malleolus and extended to a point slightly outward on the dorsum of the foot. The third strip began 5 cm above the medial malleolus and extended to a point slightly inward on the dorsum of the foot. The fourth strip was applied as a patch over the first strip.	The placebo tape application followed the same method as the test group, but without applying tension	Before taping, immediately after taping	Both KT and placebo taping were associated with improvements in SEBT performance across all directions; overall, between-condition differences were limited.
Hadadi et al. (2020)	CAIT score < 24; FAAM daily activities < 90%, FAAM sports activities < 80%; History of at least one severe ankle sprain	Strip 1: Apply at 75% stretch over the tibialis anterior muscle. Strip 2: Apply at 75% stretch around the heel. Strip 3: Apply at 75% stretch over the medial and lateral malleoli. Strip 4: Apply at 50% stretch from the navicular tubercle to the lateral malleolus.	The placebo tape application followed the same method as the test group, but without applying tension	Before taping, 4 weeks after taping	Both the KT and control taping groups showed improvements in SEBT performance over time, with only limited and direction-dependent differences between conditions.
Yin (2020)	At least one episode of lateral ankle sprain; Presence of a “giving way” sensation in the affected ankle joint, and/or recurrent sprains, and/or a feeling of instability; CAIT score < 24.	(1) With the ankle in slight dorsiflexion, the first strip originates anterior to the talus, traverses the medial and lateral malleoli, and encircles the calcaneus. (2) With the ankle in slight inversion, the second strip originates 5 cm above the medial malleolus, traverses posteriorly to the lateral malleolus, then encircles the midfoot from lateral to medial. (3) With the subject’s ankle in slight eversion, the third strip originates 5 cm above the lateral malleolus, passes posteriorly to the ankle to reach the medial malleolus, then wraps medially to the midfoot. (4) The fourth strip repeats step (1).	The placebo tape application followed the same method as the test group, but without applying tension	Before taping, immediately after taping	Compared with placebo taping, KT improved YBT anterior reach, whereas no clear between-condition differences were observed for posteromedial or posterolateral reaches.
Yu (2021)	History of two or more recurrent ankle sprains in the same ankle within the past two years; With ankle instability or loss of control during daily functional activities; CAIT score ≤ 24 on the affected side; Negative anterior drawer test on the affected side.	(1) The first tape is applied over the first cuneiform bone and first metatarsal on the dorsum of the foot, extending 10 cm to the knee. (2) The second tape is applied over the first metatarsal on the plantar aspect of the foot, extending 10 cm above the knee. (3) The third tape is applied from the medial malleolus, extending 10 cm above the knee. (4) The fourth tape is applied anterior to the lateral malleolus, extending to the plantar aspect and terminating at the transverse arch.	No intervention whatsoever	Before taping, immediately after taping	KT did not produce significant between-group differences in YBT performance compared with no taping.
Safari. et al, 2021 (Safari et al., 2023)	The participants were volleyball players with age < 30, having a history of at least a lateral ankle sprain within the past 12 months, report of at least two episodes of “giving way” feeling within the past six months, scoring ≤ 24 on CAIT, score < 90% on the daily activities section and < 80% on the sports activities section of the Persian version of FAAM questionnaire	(1)For taping the fibularis longus, an I-strip tape was used. The participants were asked to sit at the table and perform ankle inversion and plantar flexion. The tape’s origin was attached to the fibula head, and the insertion of the tape was attached to the plantar surface of the first metatarsal bone. (2)For taping the gastrocnemius muscle, two I-strip tapes were used. The participants were asked to lie in the prone position and hold ankle dorsiflexion. The origin of the first tape was attached to the medial plateau of the tibia bone. The origin of the second tape was attached to the tibia bone’s external plateau, and both tapes’ insertion was attached to the fat pad of the calcaneal bone.	The placebo tape application followed the same method as the test group, but without applying tension	Before taping, immediately after taping	KT was reported to improve YBT anterior reach compared with placebo taping, whereas findings for other outcomes were less consistent.
Wang et al. (2023)	A severe ankle sprain occurring within the past year; At least two episodes of ankle sprain or instability within six months; CAIT score ≤ 24.	(1) The first strip was placed from the dorsum of the foot around first metatarsal bones and the first cuneiform, extending upwards to tibial plateau along the anterior tibialis; (2) The second strip was placed from the first metatarsal and past the lateral malleolus, extending along the peroneus longus to the fibular head; (3) The third strip was started at the medial malleolus and extended posteriorly along the tibialis posterior to the posteromedial margin of the tibia and fibula; (4) The fourth strip was placed from the antero-lateral malleolus to the transverse arch of the foot, across the plantar aspect of the foot.	No intervention whatsoever	Before taping, immediately after taping	KT produced greater improvements than no taping in YBT anterior, posteromedial, and posterolateral reaches immediately after application.
Harry-Leite et al. (2024)	A history of at least one severe ankle sprain; At least two instances of ankle joint instability occurred; CAIT score ≤ 27 on the affected side.	Applied from the origin to insertion of the long and short peroneal (with a moderate tension of approximately 50%, to activate the muscle) and from the insertion to origin of tibialis anterior muscles (with light tension of approximately 25%, to inhibit the muscle), with the foot in plantar flexion and inversion to increase skin distension, according to the general principles of application.	The placebo tape application followed the same method as the test group, but without applying tension	Before taping, 48h after taping	In this sample of football players with CAI, KT was reported to improve YBT performance across all three directions compared with placebo taping.
Yuan et al. (2025)	A history of at least one severe lateral ankle sprain; The most recent ankle sprain occurred three months ago; CAIT score ≤ 24; The affected ankle has experienced at least two episodes of giving way or instability within the past six months.	(1) Tibialis anterior: An I-shaped strip of tape was applied from the tibial tuberosity to the front of the dorsum of the foot, covering the muscle belly of the tibialis anterior. (2) Peroneus longus: Another I-shaped strip was applied from the fibular head to just above the medial malleolus, covering the muscle belly of the peroneus longus. (3) Gastrocnemius: A Y-shaped tape was applied starting at the sole and extending to the medial and lateral epicondyles of the femur, covering the muscle belly of the gastrocnemius.	The placebo tape application followed the same method as the test group, but without applying tension	Before taping, immediately after taping	KT improved YBT anterior reach compared with placebo taping, while information on other directions was limited.

CAI, Chronic Ankle Instability; T, Test group; C, Control group; CAIT, Cumberland Ankle Instability Questionnaire; FAAM, Foot and Ankle Ability Measure; YBT, Y-Balance Test; SEBT, Star Excursion Balance Test; SHDT, Single Hop Distance Test.

### 3.3 Inclusion of literature and quality assessment results

**Figure 3:**
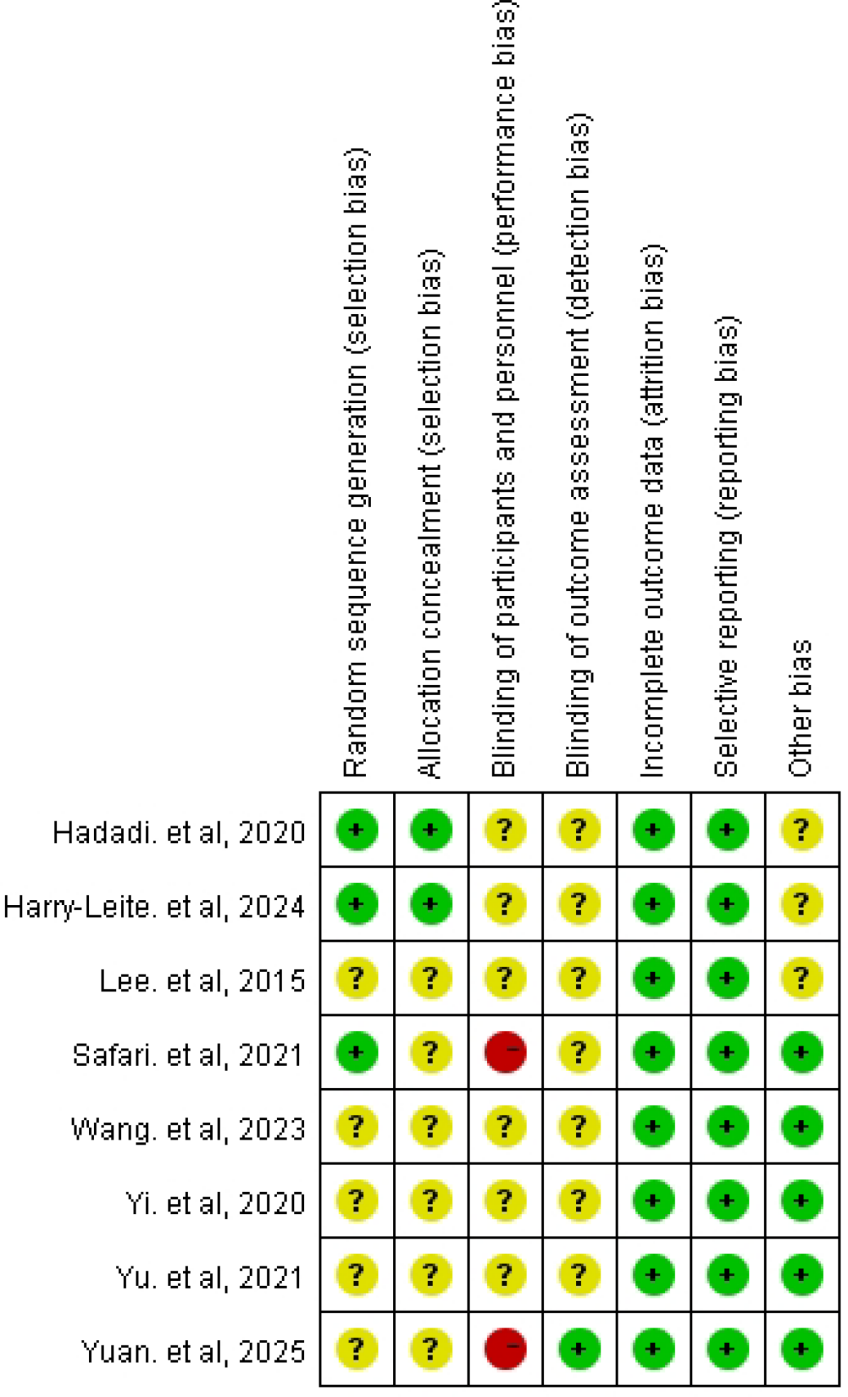
Risk-of-bias summary (traffic-light plot) for included studies.

**Figure 4:**
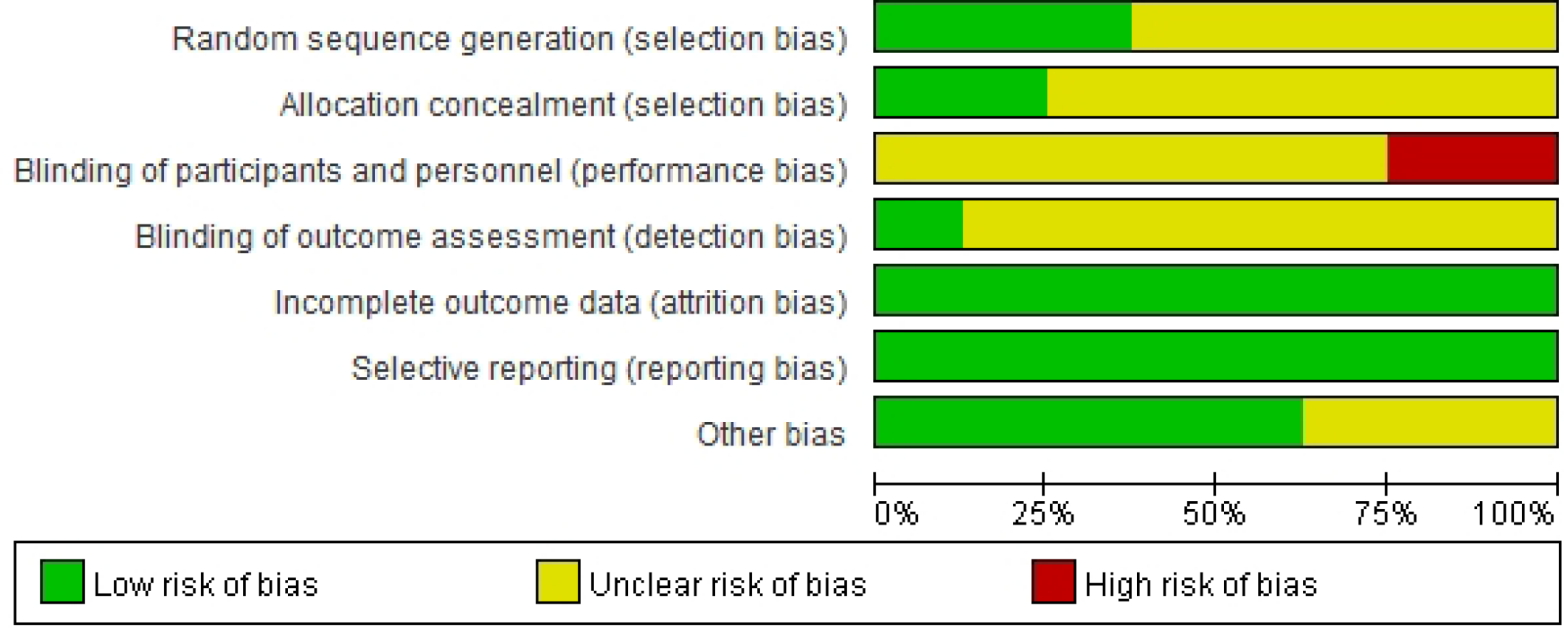
Risk-of-bias graph showing the proportion of studies rated as low, unclear, and high risk across domains.

Risk of bias was assessed using the Cochrane Risk of Bias tool (RoB 1.0). Overall, attrition bias and reporting bias were judged to be low risk in all included studies (8/8). In contrast, several key domains were frequently rated as unclear risk due to insufficient methodological reporting, particularly random sequence generation (5/8 unclear), allocation concealment (6/8 unclear), and blinding of outcome assessment (7/8 unclear). For blinding of participants and personnel, most studies were judged as unclear risk (6/8), and two studies were rated as high risk. Other potential sources of bias were generally low (5/8) but remained unclear in three studies. Detailed domain-level judgments are presented in **Figure 3**, and the distribution of risk-of-bias ratings across studies is summarized in **Figure 4**.

### 3.4 Meta-analysis results

#### 3.4.1 Y-balance test

Six studies (Yin, 2020; Yu, 2021; Safari et al., 2023; Wang et al., 2023; Harry-Leite et al., 2024; Yuan et al., 2025) reported YBT outcomes. Substantial heterogeneity was observed (*I²* = 54.5%, *P* = 0.005); therefore, a random-effects model was used. Overall, KT significantly improved YBT performance compared with the control condition (*SMD* = 0.269, *95% CI* 0.031–0.508, *P* = 0.027).

**Figure 5:**
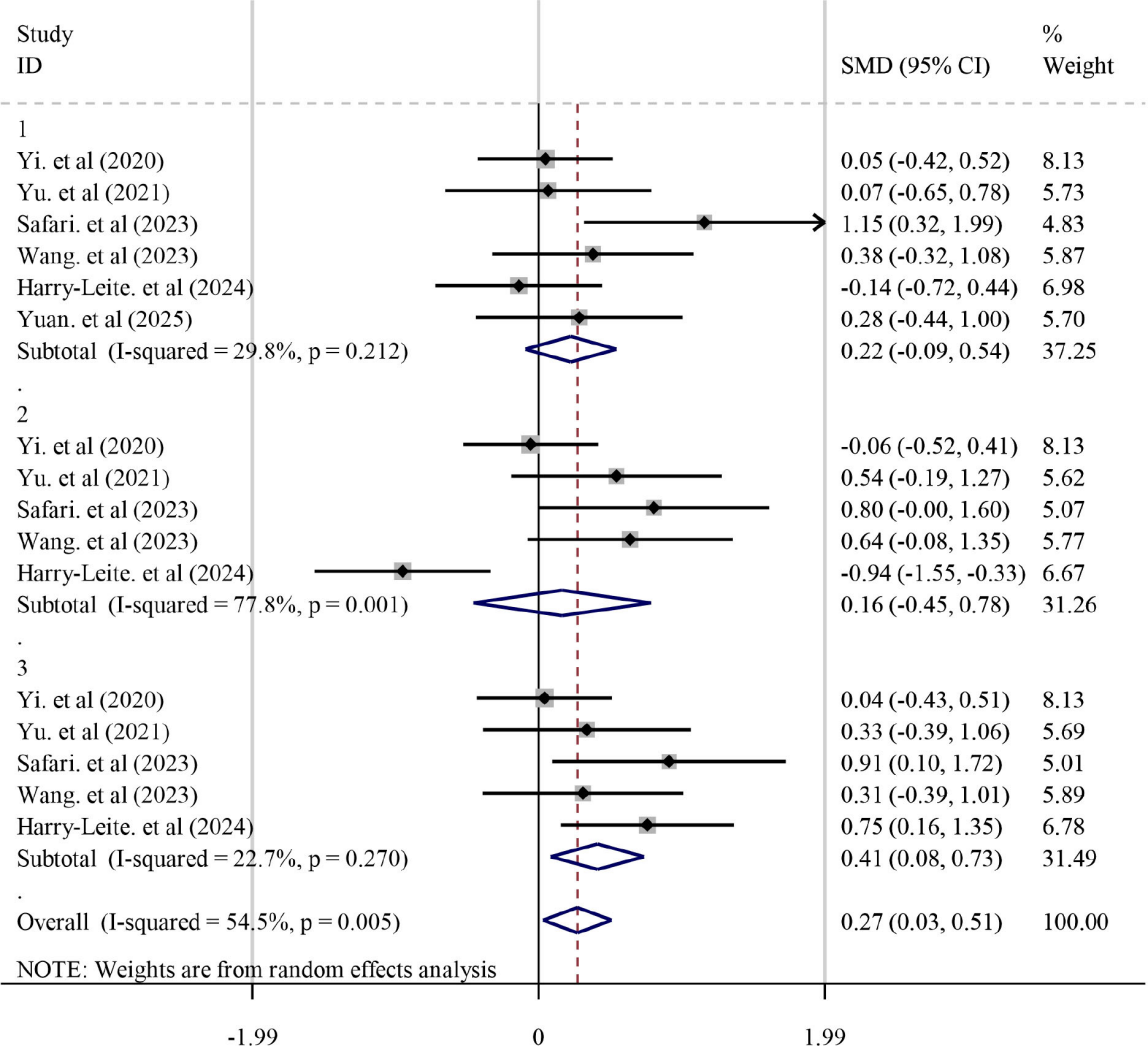
Forest plot of the effect of KT on YBT outcomes in patients with CAI. **1**: YBT anterior; **2**: YBT posteromedial; **3**: YBT posterolateral.

Because YBT includes three reach directions (anterior, posteromedial, and posterolateral), direction-specific subgroup analyses were conducted. KT significantly improved the posterolateral reach (*SMD* = 0.385, *95% CI* 0.106–0.664, *P* = 0.007), whereas effects were not significant for the anterior or posteromedial directions (both *P* > 0.05). Detailed results are presented in **Figure 5** and **Table 3**.

**Table 3:** Summary of pooled effects for YBT, SEBT, and SHDT outcomes in patients with CAI.

Subgroup	Number	Heterogeneity test results		Effect model	Results	
		*I^2^*	*p*		SMD (*95% CI*)	*P*
YBT (Yin, 2020; Yu, 2021; Safari et al., 2023; Wang et al., 2023; Harry-Leite et al., 2024; Yuan et al., 2025)	6	54.5%	0.005	Random-effects model	0.269 (0.031, 0.508)	0.027*
Direction						
YBT-anterior (Yin, 2020; Yu, 2021; Safari et al., 2023; Wang et al., 2023; Harry-Leite et al., 2024; Yuan et al., 2025)	6	29.8%	0.212	Fixed-effects model	0.195 (-0.064, 0.454)	0.140
YBT-posteromedial (Yin, 2020; Yu, 2021; Safari et al., 2023; Wang et al., 2023; Harry-Leite et al., 2024)	5	77.8%	0.001	Random-effects model	0.163 (-0.453, 0.779)	0.605
YBT-posterolateral (Yin, 2020; Yu, 2021; Safari et al., 2023; Wang et al., 2023; Harry-Leite et al., 2024)	5	22.7%	0.270	Fixed-effects model	0.385 (0.106, 0.664)	0.007*
SEBT (Lee and Lee, 2015; Hadadi et al., 2020)	2	66.1%	0.012	Random-effects model	0.509 (-0.098, 1.117)	0.100
Direction						
SEBT-anterior (Lee and Lee, 2015; Hadadi et al., 2020)	2	36.5%	0.209	Fixed-effects model	0.686 (0.083, 1.289)	0.026*
SEBT-posteromedial (Lee and Lee, 2015; Hadadi et al., 2020)	2	84.8%	0.010	Random-effects model	0.289 (-1.324, 1.092)	0.726
SEBT-posterolateral (Lee and Lee, 2015; Hadadi et al., 2020)	2	78.8%	0.030	Random-effects model	0.556 (-0.819, 1.931)	0.428
SHDT (Hadadi et al., 2020; Safari et al., 2023)	2	73.3%	0.053	Random-effects model	-0.101 (-1.156, 0.954)	0.851

YBT, Y-Balance Test; SEBT, Star Excursion Balance Test; SHDT, Single Hop Distance Test; *P < 0.05. 3.5 Publication bias.

#### 3.4.2 Star excursion balance test

Two studies (Lee and Lee, 2015; Hadadi et al., 2020) reported SEBT outcomes. Heterogeneity was considerable (*I²* = 66.1%, *P* = 0.012), and a random-effects model was applied. No significant between-group difference was found for total SEBT scores (*SMD* = 0.509, *95% CI* −0.098–1.117, *P* = 0.100).

Direction-specific analyses suggested a significant improvement in the anterior direction (*SMD* = 0.686, *95% CI* 0.083–1.289, *P* = 0.026), while posteromedial and posterolateral directions showed no significant differences (both *P* > 0.05). Given that only two studies were included, these findings should be interpreted cautiously. Results are summarized in **Table 3**.

#### 3.4.3 Single hop distance test

Two studies (Hadadi et al., 2020; Safari et al., 2023) reported SHDT outcomes. Heterogeneity was substantial (*I²* = 73.3%, *P* = 0.053), and a random-effects model was used. KT did not significantly improve SHDT compared with controls (*SMD* = −0.101, *95% CI* −1.156–0.954, *P* = 0.851). Given the small number of studies, the estimate remains imprecise. Results are presented in **Table 3**.

#### 3.4.4 Summary of meta-analysis and subgroup analysis results

Overall, pooled evidence suggests that KT provides a small improvement in dynamic balance in CAI. Subgroup analyses indicate that effects may be direction-specific, with benefits mainly observed for YBT posterolateral reach and SEBT anterior reach, whereas no significant effects were found for other directions or for SHDT. Effect estimates and confidence intervals are reported in **Table 3**.

**Figure 6:**
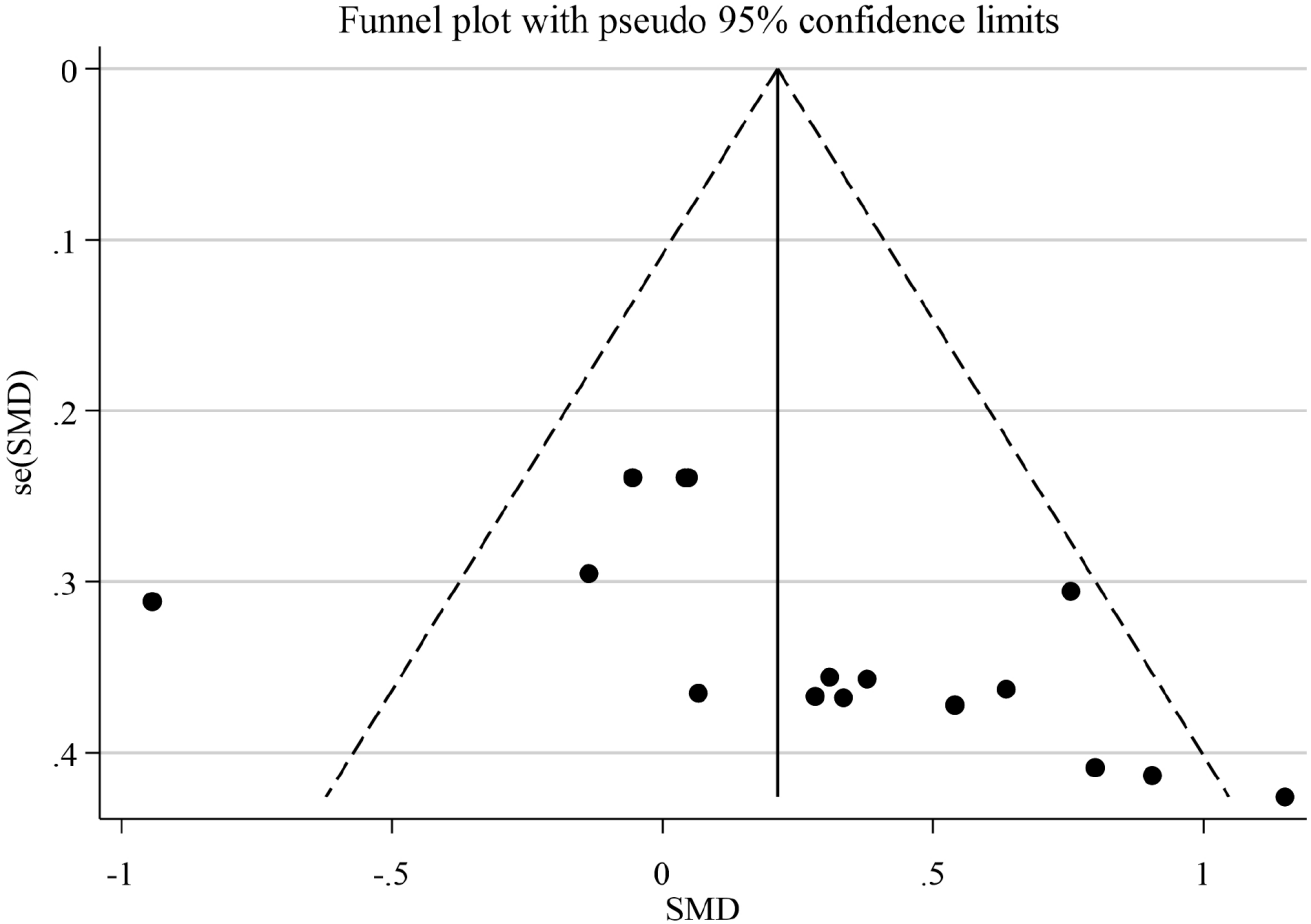
Funnel plot for the YBT outcome with pseudo 95% confidence limits.

Funnel plots were generated for the primary outcome (YBT) to explore potential publication bias (**Figure 6**). Visual inspection indicated mild asymmetry, with studies clustering on the positive effect side and relatively fewer small studies showing negative or null effects, which is consistent with possible small-study effects. However, funnel plot asymmetry is not specific to publication bias and may also reflect between-study heterogeneity or methodological differences. Given the small number of included studies (n < 10), the funnel plot has limited power to detect asymmetry; therefore, any inference should be interpreted cautiously, and formal tests (e.g., *Egger’s* or *Begg’s*) were not performed.

### 3.6 Sensitivity analysis

**Figure 7:**
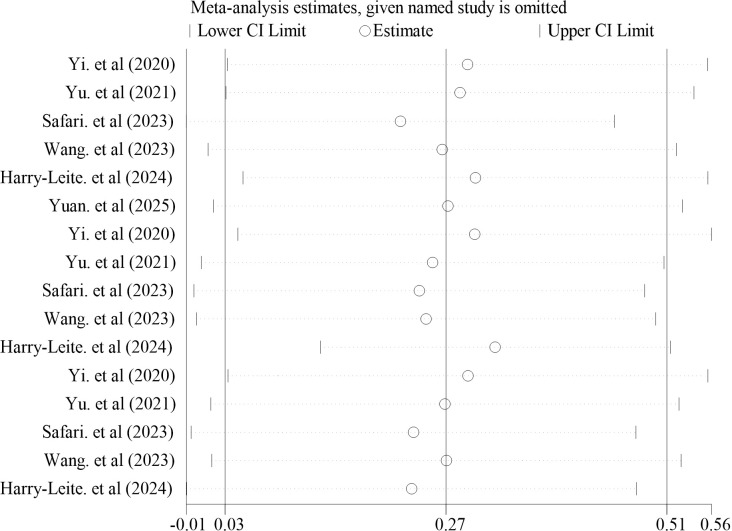
Leave-one-out sensitivity analysis for the YBT outcome.

A leave-one-out sensitivity analysis was conducted for the primary outcome (YBT). Sequential exclusion of individual studies yielded pooled effect estimates that remained close to the overall result (*SMD*≈0.27), and no single study materially altered the direction or magnitude of the pooled effect (**Figure 7**). Overall, these findings indicate that the observed improvement in YBT associated with kinesio taping is robust.

### 3.7 Certainty of evidence

Using the GRADE approach, the certainty of evidence across the included outcomes ranged from low to very low. For YBT-related outcomes, the certainty of evidence was generally low, with the exception of the posteromedial direction, which was rated as very low because of inconsistency and imprecision. For SEBT-related outcomes, the certainty ranged from low to very low: the anterior direction was rated as low, whereas the overall score and the posteromedial and posterolateral directions were rated as very low because of the small number of studies, heterogeneity, and imprecision. The certainty of evidence for SHDT was judged to be very low. Detailed GRADE ratings are presented in **Table 4**.

**Table 4:** GRADE assessment of certainty of evidence.

Outcome	Risk of bias	Inconsistency	Indirectness	Imprecision	Publication bias	Overall certainty
YBT overall	-1^①^	-1^②^	0	0	0^④^	Low
YBT-anterior	-1^①^	0	0	-1^③^	0^④^	Low
YBT-posteromedial	-1^①^	-1^②^	0	-1^③^	0^④^	Very low
YBT-posterolateral	-1^①^	0	0	-1^③^	0^④^	Low
SEBT overall	-1^①^	-1^②^	0	-1^③^	0^④^	Very low
SEBT-anterior	-1^①^	0	0	-1^③^	0^④^	Low
SEBT-posteromedial	-1^①^	-1^②^	0	-1^③^	0^④^	Very low
SEBT-posterolateral	-1^①^	-1^②^	0	-1^③^	0^④^	Very low
SHDT	-1^①^	-1^②^	0	-1^③^	0^④^	Very low

0, No downgrade; -1, Downgraded by 1 level; -2, Downgraded by 2 levels.

① The included studies had unclear or high risk in multiple key domains of RoB 1.0.

② Statistical heterogeneity and/or inconsistent directions of effect were observed across included studies.

③ The number of included studies was small and/or the 95% confidence intervals were wide, indicating imprecision.

④ Fewer than 10 studies were included; therefore, formal publication bias testing was not performed, and no downgrading for publication bias was applied in this table.

## 4 Discussion

This systematic review and meta-analysis provide updated quantitative evidence on the effects of KT on dynamic balance in individuals with CAI. Eight randomized studies (parallel-group and randomized crossover trials) were included. Compared with control conditions, KT was associated with small but statistically significant improvements in the overall YBT score and the YBT posterolateral direction, as well as the anterior direction of the SEBT. No significant effects were observed for the overall SEBT score or its posteromedial and posterolateral directions. Moreover, KT did not demonstrate a clear advantage for hop-based functional performance (SHDT) in the current evidence base. Collectively, these findings suggest that KT may offer modest, direction-specific short-term benefits for dynamic balance in CAI, while effects on broader functional performance remain uncertain. However, these findings should be interpreted cautiously because the certainty of evidence ranged from low to very low.

Several basic physiological mechanisms may help explain the observed effects of KT on dynamic balance in individuals with CAI. First, CAI itself is associated with deficits in proprioception and sensorimotor control, which may contribute to impaired postural stability and dynamic balance performance (Miklovic et al., 2018; Xue et al., 2021). KT may enhance cutaneous afferent input around the ankle and improve awareness of joint position and movement, thereby facilitating proprioceptive and sensorimotor feedback (Simon et al., 2014; Expert Consensus Group on the Clinical Application of Kinesio Taping in China, 2021; Ghai et al., 2024). In addition, KT may influence neuromuscular control during balance-related or perturbation tasks, possibly by modifying the timing or recruitment of stabilizing muscles around the ankle (Jackson et al., 2016; Shadegani et al., 2023). Although KT does not provide rigid mechanical restraint, it may also offer mild external support and improve perceived stability without substantially restricting ankle motion (Robbins et al., 1995; Expert Consensus Group on the Clinical Application of Kinesio Taping in China, 2021; Xue et al., 2022). These effects may be more relevant in specific reach directions that place greater demands on lateral stability, dorsiflexion control, or multi-planar postural adjustment, which may partly explain why the benefits observed in this review were direction-specific rather than consistent across all outcomes. However, because the included studies did not directly assess these physiological processes, this interpretation should be considered a plausible explanatory framework rather than a confirmed mechanism.

Dynamic balance is a clinically meaningful target in CAI rehabilitation because it underpins gait safety, movement quality, and the prevention of recurrent sprains (Guo et al., 2024; Tedeschi et al., 2024). Deficits in proprioception, neuromuscular control, and postural stability may directly compromise balance performance and contribute to recurrent episodes of instability (Mckeon et al., 2008; Alawna and Mohamed, 2020). From this perspective, the present findings suggest that KT may be more useful as an adjunct strategy for short-term balance-oriented rehabilitation than as a standalone intervention for broader functional recovery.

The present findings also suggest potential direction-specific effects on dynamic balance. In the YBT, KT improved the overall score and showed a more pronounced effect in the posterolateral direction. This direction may place greater demands on lateral control and weight-bearing stability and may better reflect sport-related tasks such as cutting or change-of-direction movements. In the SEBT, KT showed a statistically significant improvement in the anterior direction, while no significant differences were found in the posteromedial or posterolateral directions. The anterior reach is more dependent on ankle dorsiflexion and sagittal-plane control. Ankle dorsiflexion range of motion and sagittal-plane mechanics have been linked to gait patterns and ground reaction force regulation in CAI (Miklovic et al., 2018; Lin et al., 2021). Together, these observations may indicate that KT facilitates postural control in specific movement planes or task demands, possibly by enhancing sensory feedback and modulating neuromuscular recruitment (Robbins et al., 1995; Lee and Lee, 2017; Korkusuz et al., 2022; Shadegani et al., 2023; Yu et al., 2025). Nevertheless, the pooled effects were small, and the clinical relevance may be limited. Therefore, KT is more appropriately considered an adjunct rather than a primary intervention.

In contrast, KT did not show a significant benefit for SHDT performance. The SHDT likely reflects more complex functional capacity, requiring coordinated strength, power, and movement strategy during rapid acceleration–deceleration and landing tasks (Halim-Kertanegara et al., 2017; Alves et al., 2018; Hadadi et al., 2020). In this review, SHDT outcomes were reported by only two studies, resulting in wide confidence intervals and imprecise estimates. Thus, current evidence is insufficient to confirm that KT meaningfully improves hop-based functional performance, although potential benefits in specific populations or under particular taping protocols cannot be excluded. Overall, KT applied in isolation may have limited added value for complex functional tasks that rely heavily on strength and motor coordination. It may be more useful when integrated into comprehensive rehabilitation programs, including strengthening, proprioceptive training, and motor control or balance training (Mckeon et al., 2008; Guo et al., 2024; Tedeschi et al., 2024).

In terms of overall stability of efficacy, sensitivity analyses were conducted by sequentially excluding individual studies for YBT outcomes. Results indicated that the pooled effect size fluctuated only within a narrow range following the exclusion of individual studies, with no single study exerting a disproportionate influence on the overall conclusion. This suggests a degree of robustness in the findings. Concurrently, funnel plots for YBT exhibited a slight overall bias towards positive effect sizes. Given the limited number of included studies, funnel plots possess restricted capacity for detecting publication bias. Consequently, the possibility of small-sample effects or publication bias cannot be entirely ruled out at present, leaving the true effect of KT subject to some uncertainty.

Limitations: Several limitations of this study should be acknowledged. First, the number of studies included for each outcome was relatively small (ranging from 2 to 6), which limited statistical power and reduced the precision of pooled estimates, particularly for SEBT and SHDT outcomes. Second, variations in taping protocols (e.g., application site, tension, duration of application, and assessor timing) and differences in follow-up time points (from immediately post-intervention to several weeks later) may have contributed to clinical and methodological heterogeneity. Although we prespecified a consistent rule for time-point selection, residual heterogeneity cannot be fully excluded. Third, most included trials focused on immediate or short-term effects, whereas medium- to long-term outcomes, such as recurrence rates, sustained functional recovery, and return-to-sport performance, were rarely reported. This limited our ability to draw conclusions about the durability of KT effects. Fourth, placebo effects cannot be excluded. Sham taping or no-taping control conditions may influence participants’ perception, confidence, or protective behavior toward the ankle joint, potentially attenuating between-group differences. Fifth, several included studies used crossover designs; however, paired-difference estimates and within-subject correlation coefficients were not consistently reported. As a result, pooled analyses were based on condition-specific summary data, which may have reduced the precision of the pooled estimates and limited our ability to fully account for within-subject covariance. Finally, although the certainty of evidence was formally assessed using the GRADE framework, the overall certainty for several outcomes remained limited because of methodological concerns, small sample sizes, imprecision, and heterogeneity across studies. Accordingly, the pooled estimates should be interpreted with caution.

Future research should prioritize larger, multicenter randomized trials with rigorous randomization and improved reporting (including blinding where feasible), alongside medium- to long-term follow-up assessing clinically meaningful endpoints such as recurrence and return-to-sport. Standardized KT protocols for CAI should be developed, and comparative studies should examine the effects of taping location, tension, and application frequency on outcomes. In addition, studies should investigate potential synergistic benefits when KT is combined with structured rehabilitation programs and attempt to identify subgroups more responsive to KT using baseline clinical or biomechanical characteristics. Finally, head-to-head comparisons with other conservative options (e.g., ankle braces or rigid taping) are needed to clarify the relative role of KT within CAI rehabilitation.

## 5 Conclusion

Overall, current evidence suggests that KT may provide a small, direction-specific short-term improvement in dynamic balance in individuals with chronic ankle instability (CAI), with the clearest benefits observed in selected YBT and SEBT directions. In contrast, evidence for improvements in broader functional performance (e.g., hop performance) remains limited and imprecise. Accordingly, KT may be considered a simple adjunct to comprehensive rehabilitation programs (e.g., strengthening, proprioceptive, and balance training), but it should not be used as a stand-alone alternative to conventional interventions. Future well-designed randomized trials with standardized taping protocols, clearly reported methodology, and medium- to long-term follow-up are needed to determine the durability and clinical relevance of KT effects across different populations and task-specific contexts, and to clarify how KT can be optimally integrated with other rehabilitation modalities.

## Data Availability

All data analyzed in this study are included in the article and its **Supplementary Material**. Further inquiries can be directed to the corresponding authors.

## Supplementary material

Supplementary File 1 Full search strategies for all databases.
