# Supplementary material for: The effects of kinesio taping on dynamic balance in patients with chronic ankle instability: a systematic review and meta-analysis
Source: Front Physiol. 2026 Apr 27;17:1823162. doi: 10.3389/fphys.2026.1823162 (PMC13158095; doi:10.3389/fphys.2026.1823162)
Supplement: Supplementary File 1 — Full search strategies for all databases. [file DataSheet1.docx]

The following is the database search strategy of this study.

**Search strategy**

***P (Population):*** Participants were patients diagnosed with chronic ankle instability (CAI) as defined by the original studies, with no restrictions on sex, age, or occupation. We preferentially included studies adopting the diagnostic criteria recommended by the International Ankle Consortium, which include: (1) a history of at least one significant lateral ankle sprain; (2) recurrent episodes of “giving way” and/or perceived instability; and (3) CAI confirmed using validated functional/symptom questionnaires and/or clinical functional assessments. Studies using other diagnostic approaches were also eligible, provided that the criteria were clearly described and clinically consistent with CAI. For studies including both CAI and healthy participants, only data from the CAI cohort were extracted; healthy control data were not synthesized.

***I (Intervention):*** The experimental group received KT as the only taping intervention. Taping could be applied to structures around the ankle joint and/or lower-limb muscle groups relevant to ankle stability. Co-interventions were permitted only if applied equally to both groups.

***C (Comparison):*** The control group did not receive KT and could receive no taping or placebo/sham taping (e.g., non-tension or non-functional taping). Studies were excluded if the control group received additional functional ankle taping methods.

***O (Outcome):*** The primary outcomes were dynamic balance measures, including the Y-Balance Test (YBT) scores. Secondary outcomes included Star Excursion Balance Test (SEBT) scores and the Single Hop Distance Test (SHDT). Studies were required to report extractable quantitative data for at least one eligible outcome.

***S (Study design):*** Only randomized controlled trials (parallel-group) and randomized crossover trials were eligible. For crossover trials, randomization of condition order and an adequate washout period (or justification of no carryover) were required. Non-randomized studies, laboratory repeated-measures studies without randomization, exploratory studies, and pre–post designs without a concurrent control/sham condition were excluded.

***Exclusion criteria:*** (1) duplicate publications or studies; (2) case reports, conference abstracts, reviews, and non-randomized studies; (3) studies without a control condition, or with missing key outcome data that could not be obtained from the authors; and (4) studies with insufficient methodological information to permit risk-of-bias assessment and with no response after attempts to contact the authors.

**Search formula**

1. The search on **PubMed, Web of Science, Embase, and Cochrane Library** was performed with the following search formula:

((("chronic"[All Fields] OR "chronical"[All Fields] OR "chronically"[All Fields] OR "chronicities"[All Fields] OR "chronicity"[All Fields] OR "chronicization"[All Fields] OR "chronics"[All Fields]) AND ("ankle"[MeSH Terms] OR "ankle"[All Fields] OR "ankle joint"[MeSH Terms] OR ("ankle"[All Fields] AND "joint"[All Fields]) OR "ankle joint"[All Fields] OR "ankles"[All Fields] OR "ankle s"[All Fields]) AND ("instabilities"[All Fields] OR "instability"[All Fields] OR "instable"[All Fields])) OR (("ankle"[MeSH Terms] OR "ankle"[All Fields] OR "ankle joint"[MeSH Terms] OR ("ankle"[All Fields] AND "joint"[All Fields]) OR "ankle joint"[All Fields] OR "ankles"[All Fields] OR "ankle s"[All Fields]) AND ("instabilities"[All Fields] OR "instability"[All Fields] OR "instable"[All Fields])) OR "CAI"[All Fields]) AND (("Kinesio"[All Fields] AND ("tape s"[All Fields] OR "taped"[All Fields] OR "tapes"[All Fields] OR "taping"[All Fields] OR "tapings"[All Fields])) OR ("athletic tape"[MeSH Terms] OR ("athletic"[All Fields] AND "Tape"[All Fields]) OR "athletic tape"[All Fields] OR "kinesiotape"[All Fields] OR "kinesiotaping"[All Fields]) OR (("kinesiology zagreb"[Journal] OR "kinesiology"[All Fields]) AND "Tape"[All Fields]) OR "KT"[All Fields] OR "Tape"[All Fields])

2. The search on **CNKI, Wan Fang Data, and VIP** was performed with the following search formula:

（（慢性踝关节不稳[主题词]）或（踝关节不稳[主题词]）或（踝不稳[主题词]））和（（肌内效贴[主题词]）或（贴扎[主题词]）或（运动贴布[主题词]）或（运动机能贴[主题词]））
